# Identification of a novel Getah virus by Virus-Discovery-cDNA random amplified polymorphic DNA (RAPD)

**DOI:** 10.1186/1471-2180-12-305

**Published:** 2012-12-27

**Authors:** Tingsong Hu, Ying Zheng, Yan Zhang, Gangshan Li, Wei Qiu, Jing Yu, Qinghua Cui, Yiyin Wang, Chaoxiong Zhang, Xiaofang Zhou, Ziliang Feng, Weiguo Zhou, Quanshui Fan, Fuqiang Zhang

**Affiliations:** 1 Centre for Disease Control and Prevention, Chengdu Military Region, Kunming, 650032, China; 2 Department of Biochemistry and Molecular Biology, Fudan University Shanghai Medical College, Shanghai, 200030, China

**Keywords:** Getah virus, Identification, Virus-Discovery, cDNA RAPD

## Abstract

**Background:**

The identification of new virus strains is important for the study of infectious disease, but current (or existing) molecular biology methods are limited since the target sequence must be known to design genome-specific PCR primers. Thus, we developed a new method for the discovery of unknown viruses based on the cDNA - random amplified polymorphic DNA (cDNA-RAPD) technique. Getah virus, belonging to the family *Togaviridae* in the genus *Alphavirus*, is a mosquito-borne enveloped RNA virus that was identified using the Virus-Discovery-cDNA RAPD (VIDISCR) method.

**Results:**

A novel Getah virus was identified by VIDISCR from suckling mice exposed to mosquitoes (*Aedes albopictus*) collected in Yunnan Province, China. The non-structural protein gene, *nsP3*, the structural protein gene, the capsid protein gene, and the 3'-untranslated region (UTR) of the novel Getah virus isolate were cloned and sequenced. Nucleotide sequence identities of each gene were determined to be 97.1–99.3%, 94.9–99.4%, and 93.6–99.9%, respectively, when compared with the genomes of 10 other representative strains of Getah virus.

**Conclusions:**

The VIDISCR method was able to identify known virus isolates and a novel isolate of Getah virus from infected mice. Phylogenetic analysis indicated that the YN08 isolate was more closely related to the Hebei HB0234 strain than the YN0540 strain, and more genetically distinct from the MM2021 Malaysia primitive strain.

## Background

Viruses in the genus *Alphavirus* belong to the group IV *Togaviridae* family and include nearly 30 virus species [1]. Alphaviruses are able to infect humans and various vertebrates via arthropods, such as mosquitoes. The 11–12 kb Alphavirus genome is a single-stranded positive sense RNA flanked by a 5’ terminal cap and 3’ poly-A tail, and composed of four non-structural proteins genes (nsP1 to nsP4) and five structural proteins gene (C (nucleocapsid), E3, E2, 6 K, and E1 proteins) [2]. Getah virus (GETV) is a mosquito-borne enveloped RNA virus belonging to the Semliki Forest virus (SFV) complex in the genus *Alphavirus*[1]. To date, 10 strains of GETV have been isolated in China: M1, HB0234, HB0215-3, YN0540, YN0542, SH05-6, SH05-15–17 and GS10-2 [3]. GETV has been shown to cause illnesses in humans and livestock animals and antibodies to GETV have been detected in many animal species worldwide [4-6].

The identification of novel virus species is important for the identification and characterization of disease. However, present research methods are mostly applicable for known viruses but few methods exist to characterize unknown viruses. Current molecular biological techniques for the identification of new virus species are troublesome since some viruses do not replicate *in vitro* but some may cause a cytopathic effect. Furthermore, specific techniques that require sequence identification are not applicable. To overcome these limitations, we developed a new method for virus discovery: Virus-Discovery-cDNA RAPD (VIDISCR), based on the cDNA-random amplified polymorphic DNA technique (cDNA-RAPD) [7-11]. VIDISCR includes two key steps. First, the virus genome nucleic acid must be isolated without cellular RNA and DNA contamination. Second the RAPD analysis using the virus genome cDNA or DNA. Using this method, we tested known viruses (SV40 and SV5) and identified a new Getah virus YN08 strain. Virus nsP3, capsid protein genes, and 3’-UTR sequences were cloned, sequenced, and compared. The phylogenetic analysis indicated that the virus YN08 isolate is more closely related to Hebei HB0234 strain than the YN0540 strain, and genetically distant to the MM2021 Malaysia primitive strain.

## Results

### Virus isolation

**Figure 1:**
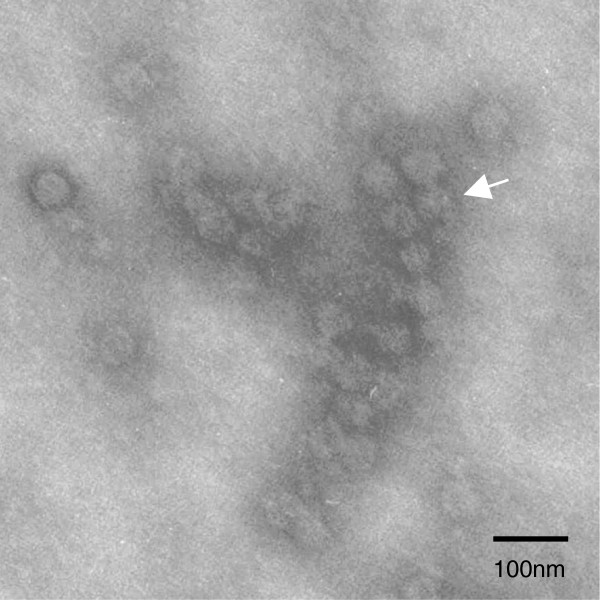
**Negatively stained electron micrograph of viral particles (arrowheads) from infected Kunming strain suckling mice brain supernatant fluid.** Bar = 100 nm.

Acute encephalitis syndrome (AES) was observed in suckling mouse with growth retardation, panting, abdominal breathing, and arthritis (data not shown). Negative-staining electron microscopy (EM) of the supernatant from infected suckling mouse brain (named YN08) revealed virus-like particles (Figure 1). These particles were spherical in shape, with an envelope, and approximately 50–70 nm in diameter, consistent in size and morphology with that of Togaviruses or Flaviviruses.

### Virus discovery using VIDISCR

**Figure 2:**
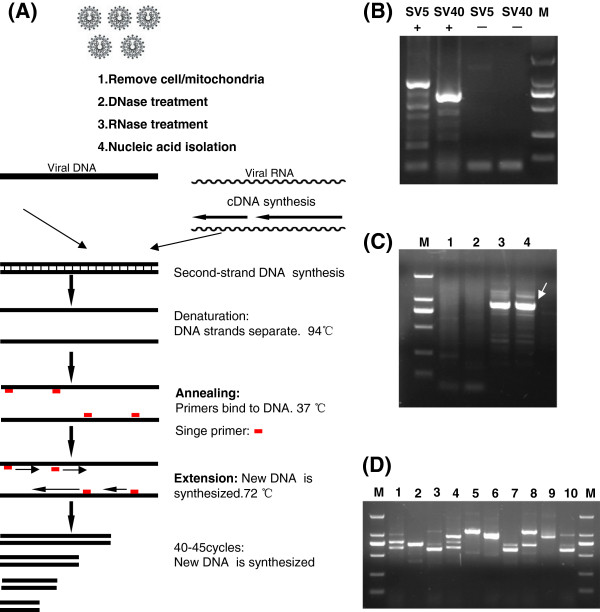
**VIDISCR method for virus identification.** (**A**) Schematic overview of steps in VIDISCR method. (**B**) Examples of VIDISCR-mediated virus identification. Specimens were analyzed using ethidium bromide-stained agarose gels (SV5 and SV40). Lane M, DNA molecular weight markers (DL2000,TOKARA); –, negative controls; +, VIDISCR PCR products for SV5 SV40 (amplified with primer S15, S14 , respectively). (**C**) VIDISCR PCR products for YN08. S11 primer was used for selective amplification; products were visualized by EB-stained agarose gel electrophoresis. Lanes 1 and 2, duplicate control supernatant from uninfected Kunming strain suckling mice; 3 and 4, duplicate PCR product of cultured YN08 harvested from brain tissues of Kunming strain suckling mice; M, DNA molecular weight markers (DL2000, Takara). Arrow indicates YN08 fragment that was excised from gel and sequenced. (**D**) VIDISCR PCR products for YN08 amplified with different primers. Lanes 1–10, PCR product of cultured YN08 amplified with different primers S1, S12, S13, S15, S21, S22, S23, S25, S38, S40, respectively; M, DNA molecular weight markers (DL2000, Takara).

The VIDISCR method was developed based on the cDNA-RAPD technique [8,9,11]. VIDISCR begins with a treatment to selectively enrich for viral nucleic acid. To remove the interferences from the cell genomes DNA and cellular RNA, a centrifugation step is used to remove residual cells and mitochondria (Figure 2A) and A DNase (and RNase) treatment is also used to remove interfering chromosomal and mitochondrial DNA (and cellular RNA) from degraded cells, where the viral nucleic acid is protected within the virus particle. The viral nucleic acids of SV40 and SV5 were detected by the VIDISCR method (Figure 2B) from cell culture, demonstrating its capacity to identify both DNA and RNA viruses (Figure 2B and Table 1).

**Table 1:** RAPD Primers used for VIDISCR and the result of Virus discovery by the VIDISCR method

**Primer**	**Sequence (5’-3’)**	**SV5**	**SV40**	**YN08**
S1	GTTTCGCTCC	N	N^*^	2/3
S2	TGATCCCTGG	N	1/3^*^	N
S3	CATCCCCCTG	2/2	N	N
S4	GGACTGGAGT	1/3	N	N
S5	TGCGCCCTTC	N	1/2	N
S11	GTAGACCCGT	1/3	N	1/1
S12	CCTTGACGCA	2/3	1/1	1/2
S13	TTCCCCCGCT	N	N	1/2
S14	TCCGCTCTGG	1/1	1/2	N
S15	GGAGGGTGTT	2/3	N	2/2
S21	CAGGCCCTTC	N	N	2/2
S22	TGCCGAGCTG	N	N	1/2
S23	AGTCAGCCAC	1/3	N	1/2
S24	AATCGGGCTG	N	N	N
S25	AGGGGTCTTG	N	0/2	1/2
S36	AGCCAGCGAA	2/4	N	N
S37	GACCGCTTGT	1/1	N	N
S38	AGGTGACCGT	N	N	0/1
S39	CAAACGTCGG	N	1/2	N
S40	GTTGCGATCC	N	N	1/2

*Note: “N” denote The unique and prominent DNA fragments were not present in the test sample ; the denominator was the number of The unique and prominent DNA fragments by cloned and the numerator was the number of the virus DNA fragments in the test sample.

The supernatant of the suckling mouse brain tissue infected with YN08 was analyzed by VIDISCR. The supernatant of uninfected suckling mouse brain tissue was used as a negative control. Unique amplified DNA fragments were present in the test sample but not in the control where the 11 reactions gave prominent DNA fragments in 20 VIDISCR selective PCR reactions (11/20 selective PCR; Figure 2C & D, Table 1). The 21 VIDISCR fragments were cloned and sequenced from the 11 selective PCR assays. Thirteen of 21 fragments showed sequence similarity to members of the *Togaviridae* family with 98% identity to GETV using the basic local alignment search tool (BLAST).

### PCR amplification, sequence analysis, and phylogenetic comparisons

Using VIDISCR, the non-structural protein gene nsP3, the structural protein gene capsid protein gene and 3’-UTR sequences of the YN08 isolate were amplified, cloned, and sequenced. Other GETVs non-structural protein genes nsP3, capsid protein genes and 3’-UTR sequences obtained from databases were compared, including those from MM2021 (Malaysia), MAG (Russia), ALPV_M1, (China) GETV_M1 (China), MPR (Mongolia), S_KOREA (South Korea), HB0234 (China Hebei, China), YN0540 (Yunan, China), and SAGV (Sagiyama virus from Japan). The YN08 isolate non-structural protein gene nsP3, the structural protein gene (capsid protein gene), and 3’-UTR sequence identity were 97.1–99.3%, 94.9–99.4%, and 93.6–99.9%, respectively, by alignment with 10 strains of Getah virus found worldwide. Analysis of all sequences (nsP3, capsid protein gene, and 3’-UTR) included in this study showed the highest nucleotide sequence identity between YN08 and GETV HB0234 strains.

The YN08 isolate nsP3 nucleotide sequences identity ranged from 98.00 to 99.31%, while amino acid sequence identity ranged from 98.89 to 99.44% (Table 2) between YN08 isolates and other Chinese isolates (GETV_M1[12], ALPV_M1 HB0234, and YN0540). The capsid protein gene nucleotide sequence identity ranged from 97.56 to 99.31%, while amino acid sequence identity ranged from 98.27 to 99.66% (Table 3) between YN08 isolates and other Chinese isolates (GETV_M1 [12], ALPV_M1 HB0234 and YN0540).

**Table 2:** Homology comparison of nucleotide (below the diagonal) and amino acid sequences (above the diagonal) of non-structural protein gene nsP3 of YN08 isolates Getah virus with other Alphavirus isolates

	**1**	**2**	**3**	**4**	**5**	**6**	**7**	**8**	**9**
**1.** AlpV_M1		99.07%	98.89%	98.89%	99.07%	100%	**98.89%**	98.70%	99.07%
**2.** GETV_S_Korea	98.4%		99.63%	99.07%	99.63%	99.07%	**99.82%**	99.44%	98.89%
**3.** GETV_HB0234	98.1%	99.4%		98.89%	99.26%	98.89%	**99.44%**	99.44%	98.70%
**4.** GETV_LEIV_16275_MAG	97.9%	97.4%	97.2%		99.07%	98.89%	**98.89%**	98.70%	99.07%
**5.** GETV_LEIV_17741_MPR	98.6%	98.8%	98.5%	97.9%		99.07%	**99.44%**	99.07%	98.89%
**6.** GETV_M1	99.9%	98.5%	98.2%	98.0%	98.7%		**98.89%**	98.70%	99.07%
**7. GETV_YN08**	**98.0%**	**99.3%**	**99.3%**	**97.1%**	**98.3%**	**98.1%**		**99.26%**	**98.70%**
**8.** GETV_YN0540	98.1%	99.4%	99.1%	97.2%	98.5%	98.2%	**99.0%**		98.51%
**9.** SAGV	98.1%	97.5%	97.2%	98.5%	97.9%	98.2%	**97.1%**	97.2%	

**Table 3:** **Homology comparison of nucleotide and amino acid sequences of Capsid gene of YN08 isolates Getah virus with other Alphavirus isolates**^**a**^

	**1**	**2**	**3**	**4**	**5**	**6**	**7**	**8**	**9**	**10**
**1.** ALPV_M1		99.66%	99.66%	99.66%	98.97%	97.57%	99.66%	**99.31%**	99.66%	99.31%
**2.** GETV_HB0234	98.50%		99.31%	100%	98.62%	97.22%	100%	**99.66%**	100%	98.97%
**3.** GETV_LEIV_16275_Mag	98.85%	97.79%		99.31%	98.62%	97.22%	99.31%	**98.97%**	99.31%	98.97%
**4.** GETV_LEIV_17741_MPR	99.20%	98.85%	98.27%		98.62%	97.22%	100%	**99.66%**	100%	98.97%
**5.** GETV_M1	99.67%	98.15%	98.50%	98.85%		96.51%	98.62%	**98.27%**	98.62%	98.27%
**6.** GETV_MM2021	96.25%	95.14%	95.90%	95.64%	95.88%		97.22%	**96.87%**	97.22%	97.57%
**7.** GETV_S_Korea	98.62%	99.66%	97.91%	98.97%	98.27%	95.27%		**99.66%**	100%	98.97%
**8.** GETV_YN08	**98.27%**	**99.31%**	**97.56%**	**98.62%**	**97.91%**	**94.89%**	**99.43%**		**99.66%**	**98.62%**
**9.** GETV_YN0540	98.50%	99.32%	97.80%	98.86%	98.15%	95.15%	99.43%	**99.08%**		98.97%
**10.**SAGV	98.03%	97.2%	98.04%	97.68%	97.68%	96.50%	97.32%	**96.96%**	97.44%	

Note: ^**a**^ The lower left part represents the homologous rate of nucleotide sequence of viral Capsid gene The upper right part represents the homologous rate of amino acid sequence of viral Capsid gene.

Alphaviruses possess a highly conserved 3’ sequence element (3’ CSE; approximately 19 nt long) that immediately precedes the poly(A) tail [2]. Both the poly(A) tail and the 3’CSE are required for virus replication and, more specifically, for efficient minus-strand RNA synthesis [13-17]. The terminal 19 nt conserved sequence was identical in all GETV isolates, including the M1 isolate that was previously reported to have lost this conserved sequence [18,19].

Alignment with the other nine strains of Getah virus indicated that the 3’-UTR sequence homology between YN08 isolate and other Chinese isolates (GETV_M1, ALPV_M1, HB0234 and YN0540) ranged from 99.65 to 99.77% (Table 4). Analysis of all 3’-UTR sequences included in this study showed the highest nucleotide sequence identity between the YN08 isolate and MPR GETV (99.89%) and the nucleotide sequence identity was lowest between the YN08 isolate and the South Korean isolate (93.61%).

**Table 4:** Percent Identity (below the diagonal) and Divergence (above the diagonal) matrix of 3' UTR sequence of different Alphavirus isolates

	**1**	**2**	**3**	**4**	**5**	**6**	**7**	**8**	**9**	**10**
**1.** ALPV_M1		0.0035	0.0046	0.0023	0.0011	0.0118	0.0626	**0.0035**	0.0035	0.0011
**2.** GETV_HB0234	99.65%		0.0082	0.0012	0.0023	0.0155	0.0640	**0.0023**	0.0023	0.0046
**3.** GETV_LEIV_16275_Mag	99.54%	99.18%		0.0070	0.0058	0.0142	0.0656	**0.0082**	0.0082	0.0058
**4.** GETV_LEIV_17741_MPR	99.77%	99.88%	99.30%		0.0012	0.0143	0.0625	**0.0011**	0.0012	0.0035
**5.** GETV_M1	99.89%	99.77%	99.42%	99.88%		0.0130	0.0641	**0.0023**	0.0023	0.0023
**6.** GETV_MM2021	98.82%	98.45%	98.58%	98.57%	98.70%		0.0781	**0.0155**	0.0155	0.0130
**7.** GETV_S_Korea	93.74%	93.60%	93.44%	93.75%	93.59%	92.19%		**0.0639**	0.0640	0.0626
**8.** GETV_YN08	**99.65%**	**99.77%**	**99.18%**	**99.89%**	**99.77%**	**98.45%**	**93.61%**		**0.0023**	**0.0046**
**9.** GETV_YN0540	99.65%	99.77%	99.18%	99.88%	99.77%	98.45%	93.60%	**99.77%**		0.0046
**10.**SAGV(DNA)	99.89%	99.54%	99.42%	99.65%	99.77%	98.70%	93.74	**99.54%**	99.54%	

### Phylogenetic analysis

**Figure 3:**
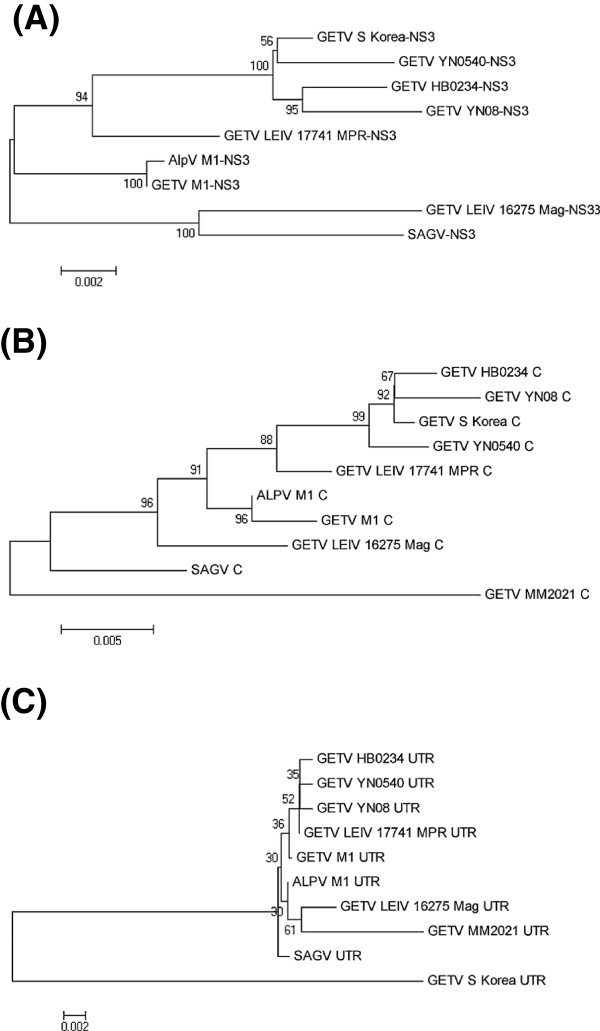
**Phylogenetic relationship betweenYN08 isolates of GETV and other alphaviruses based on the non-structural protein gene nsP3, capsid protein and 3' UTR area sequences.** The neighbor joining tree was constructed using the MEGA with bootstrapping. (**A**) Phylogenetic analysis of RT-PCR sequences of the non-structural protein gene nsP3 from YN08 isolates of GETV and other alphaviruses. (**B**) Phylogenetic tree constructed using the nucleotide sequences of the capsid gene of YN08 isolates of GETV and other alphaviruses. (**C**) Phylogenetic tree constructed using the nucleotide sequences of 3’-UTR area sequences of GETV isolates.

To better understand the genetic relationship of YN08 to other strains of Getah virus in the world (including Chinese isolates ALPV_M1, GETV_M1, HB0234, and YN0540), the previously published genetic sequences of GETV and other alphavirus capsid protein genes and 3’-UTR sequences obtained from GenBank were used to construct phylogenetic trees. The phylogenetic analyses clearly showed that YN08 is more closely related to the Hebei HB0234 strain than the YN0540 strain, and more distantly related to the MM2021 Malaysia primitive strain (Figure 3).

## Discussion

Alphaviruses are mosquito-borne RNA viruses that cause devastating or debilitating diseases in both humans and livestock. SAGV and GETV are two members of the *Alphavirus* genus of the family *Togaviridae*. GETV is widely distributed in southeast Asia and northern Australia along the Pacific Ocean [20-24]. GETV has been isolated from various mosquito species of the genera *Culex*, *Aedes*, and *Armigeres*[18]. It is conceivable that GETV may use mammals as primary hosts in its enzootic cycle, and through these biting vectors, the virus may be transmitted to various animal species, including pigs, chickens, humans, and other mammals, on rare occasion, the virus may jump the species barrier and infect a human or other animal [25]. Although the virus has not been linked to illness in humans, many studies have suggested that the virus is a latent pathogen of humans causing a fever of unknown origin. GETV could cause illnesses in humans and livestock animals and, indeed, antibodies to GETV have been detected in many species of animals around the world [4-6].

Analysis of all sequences included in this study showed that the nsP3 non-structural protein gene and the capsid protein gene nucleotide sequence identity between YN08 isolates and other Chinese isolates (GETV_M1 [12], ALPV_M1, HB0234 and YN0540) ranged from 98.0 to 99.31% and 97.56 to 99.31%, respectively. Multiple alignments showed that the S_Korea isolate does not possess the 92 nt sequence from 11341–11433 in the virus genome and there was a low level of identity (92.19–93.75%) between S_Korea and other GETV strain at the 3’-UTR sequences. Despite possessing 3’-UTR sequences of different lengths, GETV isolates contain various numbers of an identical sequence element that could have originated from a large ancestral 3’-UTR [26,27].

Phylogenetic trees constructed using viruses sequence data are the best indication of the evolutionary relationships between viruses and genetic changes associated with antigenic drift. To provide further insight into the evolutionary relationship of YN08 and other alphaviruses, phylogenic analysis was performed based on the capsid protein gene and the 3’-UTR sequence of YN08 and other 9 alphaviruses. These analyses showed that YN08 is a member of the GETV and was most closely related to HB0234 and S_Korea and then with YN0540 and GETV_LEIV_17741_MPR to form a distinguishable branch based on nsP3 and capsid protein genes. Thus, the phylogenetic analysis clearly showed that YN08 is more closely related to Hebei HB0234 strain than YN0540 strain and more genetically distant to the MM2021 Malaysia primitive strain.

Present methods rely on prior genetic knowledge but are not effective for the identification of unknown viruses. Thus, we developed the simple VIDISCR method based on the cDNA-RAPD technique [8,9]. The RAPD technique is a type of PCR but random segments of DNA are amplified. Unlike traditional PCR analysis, RAPD does not require any specific knowledge of the DNA sequence of the target organism by the use of 10-mer primers for the amplification of DNA. However, the resolving power of the VIDISCR method is prone to interference from DNA or RNA from the lysed host tissues and cells (or bacteria). Since VIDISCR relies on a large, intact DNA template sequence, it has some limitations in the use of degraded DNA samples. Therefore, the intact DNA template sequence of virus genomes required and chromosomal DNA, mitochondrial DNA, and cellular RNA must be removed from the preparation to perform VIDISCR. In the current study, approximately 50% of PCR assays amplified DNA fragments in 20 VIDISCR selective PCR reactions (11/20; Table 1) and 13 of 21 fragments showed sequence similarity to virus genes. Therefore, VIDISCR is a suitable method for the identification of unknown viruses.

The current study indicated that the VIDISCR is an efficient procedure for the identification of known and unknown viruses with the removal of contaminating cellular nucleic acids, optimized nucleic acid amplification, large-scale sequencing, and bioinformatics. The VIDISCR technology is general, non-selective, and rapid, that does not require prior knowledge of the target sequence. This technique could be adapted to include a set of universal primers for virus genomic analysis in a wide variety of species. VIDISCR can identify a range of known and unknown pathogens that can be applicable to clinical samples including tissues or culture supernatants. Therefore, it is well suited for the rapid identification of an unknown or unexpected virus involved in a disease outbreak.

## Conclusions

The present study described the isolation and identification of a new Getah virus YN08 with the VIDISCR method. Phylogenetic analysis indicated that the virus YN08 isolate was more closely related to Hebei HB0234 strain than YN0540 strain, and the virus was distantly related to the MM2021 Malaysia primitive strain. This study provided a VIDISCR method based on the cDNA-RAPD technique that is well suited for rapid identification of known and unknown or unexpected viruses involved in a disease outbreak.

## Methods

### Mosquito collection, treatment, and virus isolation

Mosquitoes were collected from villages where livestock were bred in Yunnan province in 2008. Collection locations were within 10 m of henhouses, hog pens, and sheep pens. Collected mosquitoes were frozen for 30 min at −20°C and then placed on an ice plate to determine mosquito species and to exclude blood-fed or male mosquitoes. Fifty to 100 mosquitoes were sorted into a collection tube and stored in liquid nitrogen. Pooled mosquitoes were added to 2 mL minimal essential medium (MEM, HyClone Laboratories, Inc. 925 West 1800 South Logan, Utah 84321) supplemented with 2 mM glutamine, 0.12% NaHCO_3_, 100 U/mL penicillin, and 100 U/mL streptomycin, followed by grinding in a pre-cooled sterile plastic grinding tube. The ground samples were centrifuged at 13 800 × g in a microcentrifuge for 20 min at 4°C. Virus isolation was attempted in suckling mouse brain by injecting 20 μL of clarified supernatant in the capsule of brain of 2–3 day old Kunming mice. The use of animals complied with the guidelines of the Experimental Animal Ethics Committees of the Centre for Disease Control and Prevention, Chengdu Military Region.

### VIDISCR

Virus controls, including SV40 and SV5, were cultured on Vero E6 cells. Culture supernatants of SV40 and SV5 viruses were analyzed by VIDISCR to assess the general applicability of the technique. The unknown (YN08) virus was cultured in the capsule of brain of 2–3 day old Kunming suckling mice. Pooled brain tissues contai-ning virus were added to 2 mL MEM, followed by homogenization in a pre-cooled sterile plastic grinding tube. To remove residual cells and mitochondria, 110 μL brain homogenate supernatant was centrifuges for 10 min at maximum speed (17 000 × g) in a microcentrifuge at 4°C. To remove chromosomal DNA and mitochondrial DNA from the lysed cells, 100 μL of supernatant was transferred to a fresh tube and treated with DNase I for 45 min at 37°C (Takara) [7,8]. To remove host RNA from the preparation, the supernatant was treated with RNase A (Takara) for 5 min at 37°C. Nucleic acids were extracted using the AxyPrep Body Fluid Viral DNA/RNA Miniprep Kit (Axygen, Inc.) [28]. The ribonuclease inhibitor is required to obtain the intact RNA sequence of virus genomes.

A reverse transcription reaction was performed with random hexamer primers (Takara) and Moloney murine leukemia virus reverse transcriptase (MMLV-RT; Invitrogen). Second-strand DNA synthesis was carried out using Sequenase II (Takara) without further addition of primers. A phenol-chloroform extraction was followed by ethanol precipitation. The cDNA-RAPD assay was performed as previously described [9-11], with some modifications. The PCR program commonly used for RAPD analysis with random 10-mer primers (Table 1) included a 30-s template denaturing step at 94°C, a 30-s primer annealing step at 37°C and a 1-min primer extension step at 72°C. RAPD primers were purchased from Sangon Biotech (Shanghai, China) and consisted of 2160 primers named from S1 to S2160 and for the current assay, 20 primers were chosen from the S1 to S40 subset. Thermocycling typically consisted of 45 cycles of these three steps to obtain a RAPD pattern. The PCR products were analyzed on ethidium bromide (EB)-stained 2% agarose gels and the amplified fragments of interest were cloned and sequenced using BigDye terminator reagents. Electrophoresis and data collection were performed using an ABI 377 instrument (ABI). DNA molecular weight markers were obtained from Takara.

### Identification of virus by electron microscopy

GETV was observed by EM. Preparation of the sample from a 1/10 volume of the brain extract from suckling mice included extraction with chloroform and incubation of the mixture for 30 min at 4°C. The extract was then centrifuged at 13 800 × g for 30 min. The precipitate was resuspended in 5 mM phosphate buffered saline (PBS; pH 7.2) and negatively stained with 2% phosphotungstic acid. Specimens were examined using a transmission electron microscope (Hitachi-8100, Japan) at 80 kV.

### PCR amplification and sequencing

The Getah virus nsP3 non-structural protein gene, the structural protein gene capsid gene and 3’-UTR primer sequences used were as follows [29]: nsP3 gene sense primer NS3-S: 5’-ATG CCT GCA ACG GAT TGC-3’, antisense primer NS3-R: 5’-CGG GCC AGT GTC AGA CG-3’; capsid gene sense primer GETC1: 5’-CAG GAT TAC ACT ACA TCT AAA G-3’, antisense primer GETC2: 5’-ACG TTG GCT AAG ACG CAC ATC-3’; 3’-UTR sense primer GETU1: 5’-CGG CAA T GA CAT GGG TGC AGC-3’ antisense primer GETU2: 5’-CTG TCA GCG AAT TCG GTA CTT TTT TTT TTT TTT TTT TG-3’. PCR conditions were 94°C for 3 min, followed by 40 cycles of DNA amplification (45 s at 94°C, 1 min at 61°C, and 1 min 30 s at 72°C) and 8 min incubation at 72°C. PCR products were analyzed on 1.2% (w/v) agarose gels by electrophoresis at a constant voltage (2 V/cm). The non-structural protein gene nsP3, the capsid proteins genes and 3’-UTR sequences were cloned and sequenced.

### Sequence analysis and phylogenetic comparisons

Sequence data were analyzed using computer programs such as DNAMAN and DNASTAR. Phylogenetic analyses were performed by the neighbor-joining method using MEGA (version 5.05; http://www.megasoftware.net/). Previously published GETV sequences used in this study include sequences YN08 isolates MM2021 (from Malaysia, GenBank:AF339484), MAG (from Russia, EF631998), ALPV_M1 x(from China, EF011023), GETV_M1 (from China Hainan, EU015061), MPR (MPR from Mongolia, EF631999), S_KOREA (from South Korea, AY702913), HB0234(from China Hebei, EU015062), YN0540 (from China Yunan, EU015063), and SAGV (Sagiyama virus from Japan, AB032553).

## Competing interests

The authors declare that they have no competing interests.

## Authors' contributions

Tingsong Hu, Ying Zheng and Yan Zhang participated in the design and conducted the majority of the experiments in the study and drafted the manuscript. Gangshan Li, Wei Qiu and Jing Yu carried out the molecular genetic studies, participated in the sequence alignment. Qinghua Cui,Yiyin Wang, Caoxiong Zhang and Xiaofang Zhou contributed to the interpretation of the findings and revised the manuscript. Ziliang Feng and Weiguo Zhou performed the analyses of transmission electron microscope. Quanshui Fan and Fuqiang Zhang participated in the design of the study and performed the statistical analysis. All authors read and approved the final manuscript.
